# Supplementary figures and images for: EpiCurator: an immunoinformatic workflow to predict and prioritize SARS-CoV-2 epitopes
Source: PeerJ. 2021 Nov 30;9:e12548. doi: 10.7717/peerj.12548 (PMC8641484; doi:10.7717/peerj.12548)

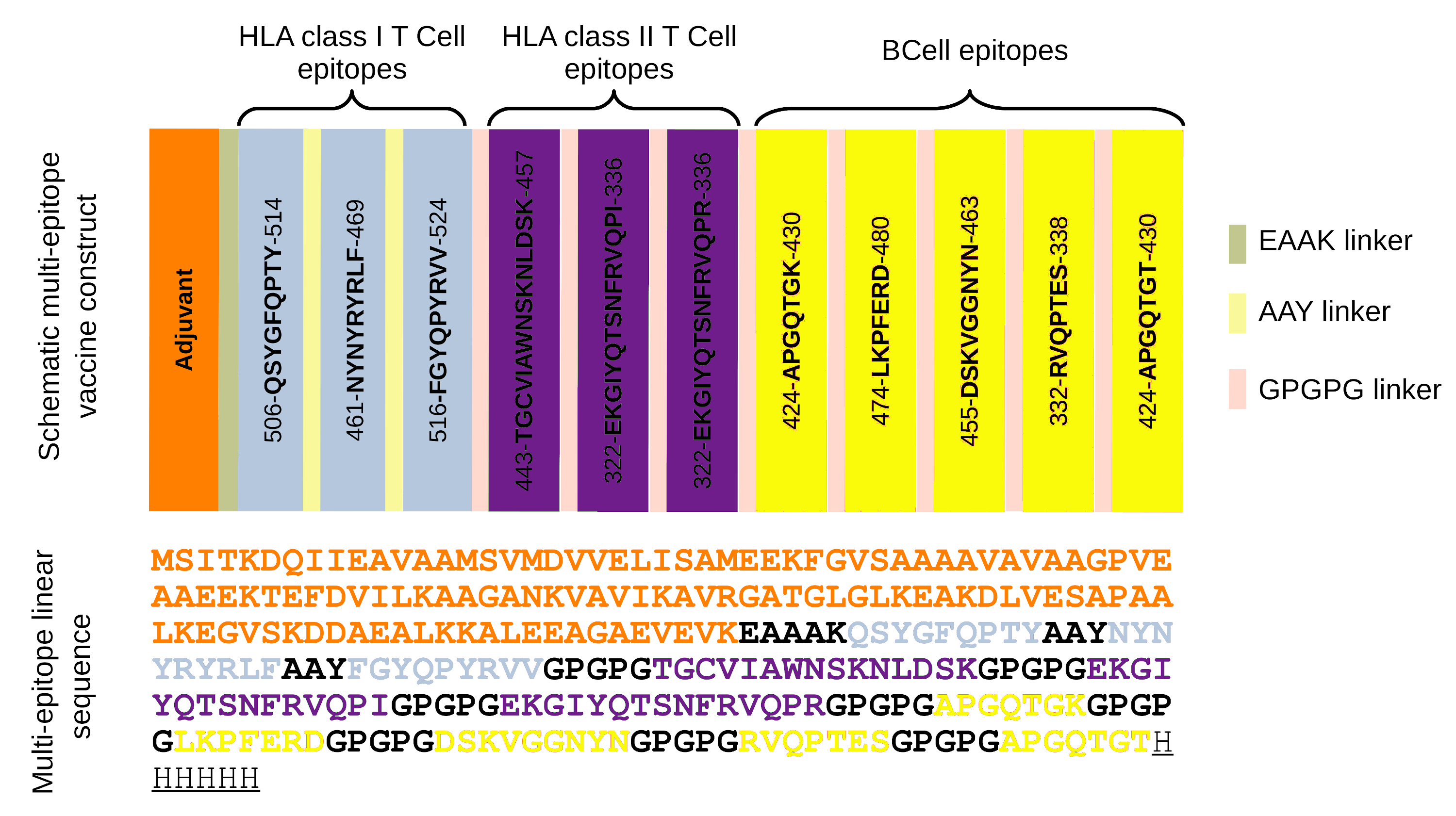

Supplement: Supplemental Information 1 — The multi-epitope structure is constructed by 11 subunits (blue subunits represents HLA class I epitopes, purple subunits represents HLA class II epitopes and yellow subunits represents B-cell epitopes), an adjuvant represented in orange, linked by EAAAK, AAY and GPGPG linkers (right legend). The linear sequence of the multi-epitopes is represented with the same colors previously listed, added with a histidine hexamer. [file peerj-09-12548-s001.png]

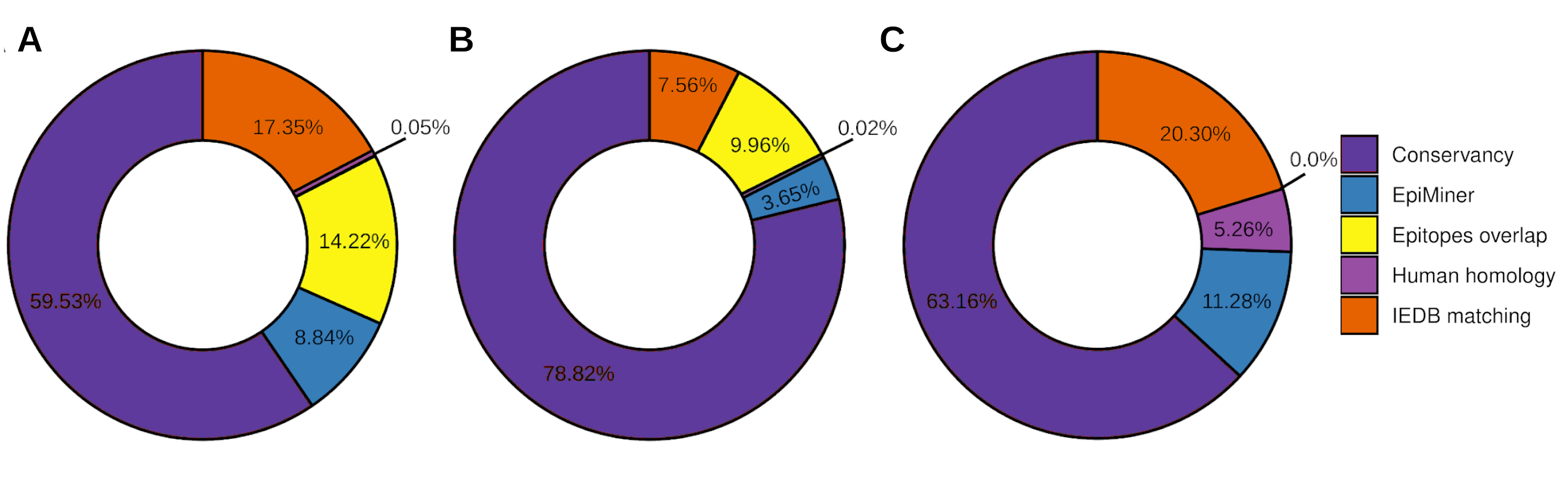

Supplement: Supplemental Information 2 — The plot represents the percentage of epitopes removed and each different analysis identified in the caption for the HLA class I epitopes (A), HLA class II epitopes (B) and B cell epitopes (C). [file peerj-09-12548-s002.png]

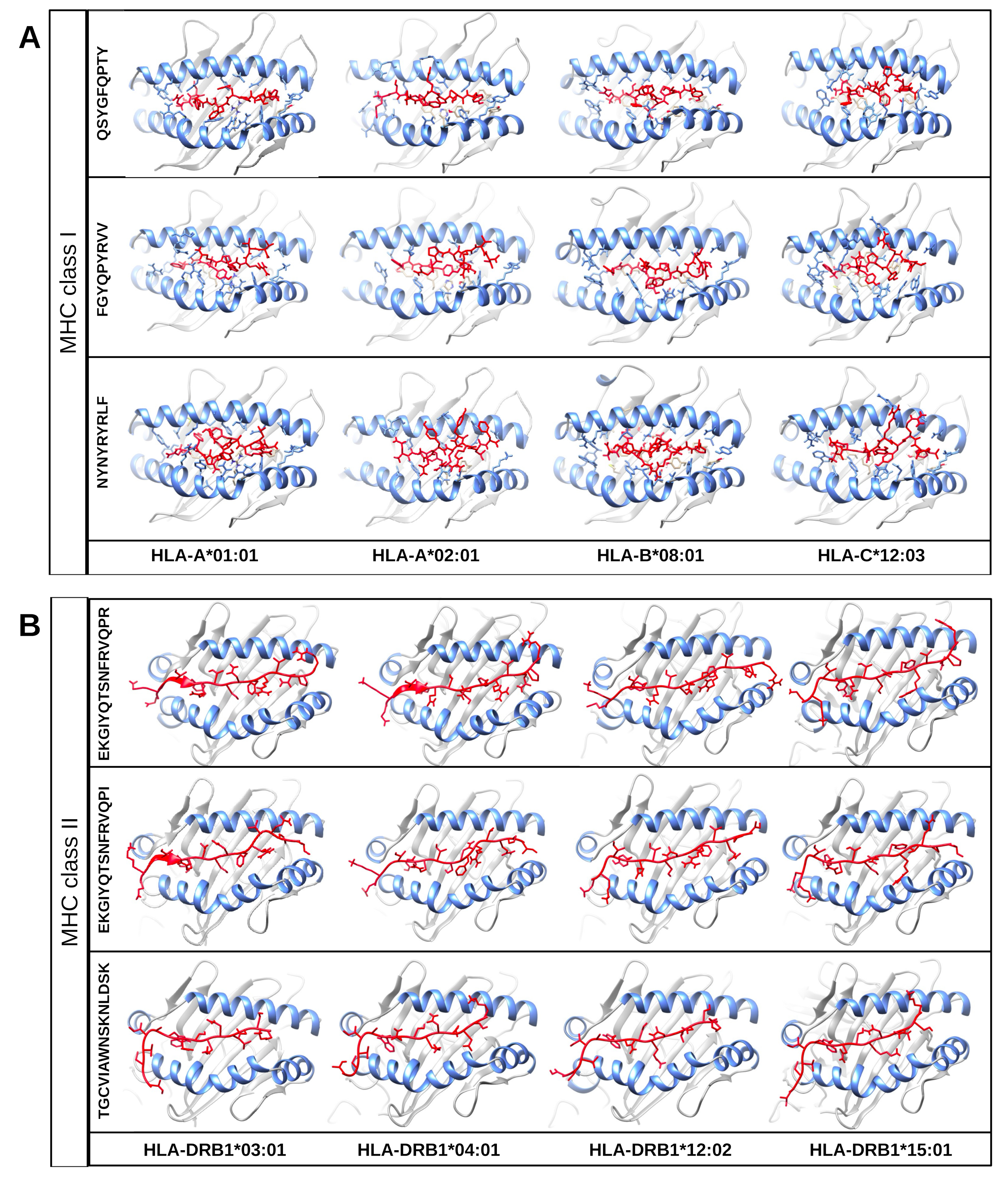

Supplement: Supplemental Information 3 — Structure complexes provided by docking simulation show the MHC binding grooves (blue ribbons), and the epitope (red structure) for HLA class I alleles (A) and HLA class II alleles (B). For each complex, the amino acids sequence of the epitope and HLA binding allele are available. [file peerj-09-12548-s003.png]

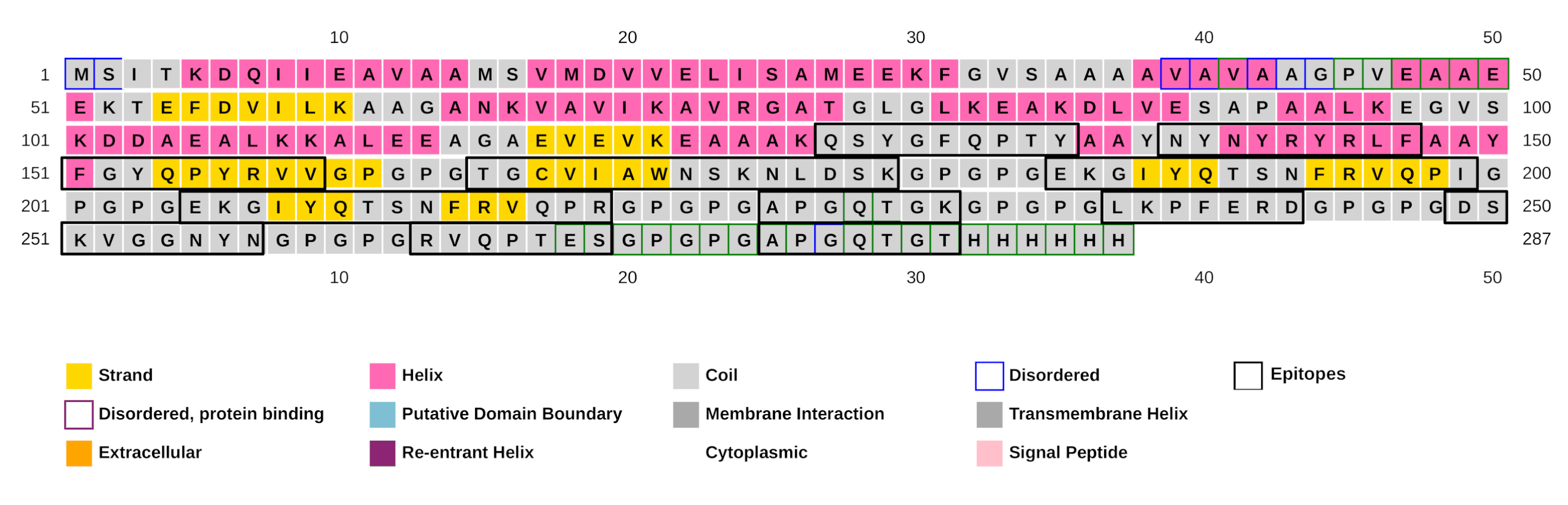

Supplement: Supplemental Information 4 — The alpha helix residues are in pink square, the beta strand residues are in yellow square, the coil residues are in grey square. The disordered residues are in Blue border square and purple border square and the epitopes are in black border square. [file peerj-09-12548-s004.png]

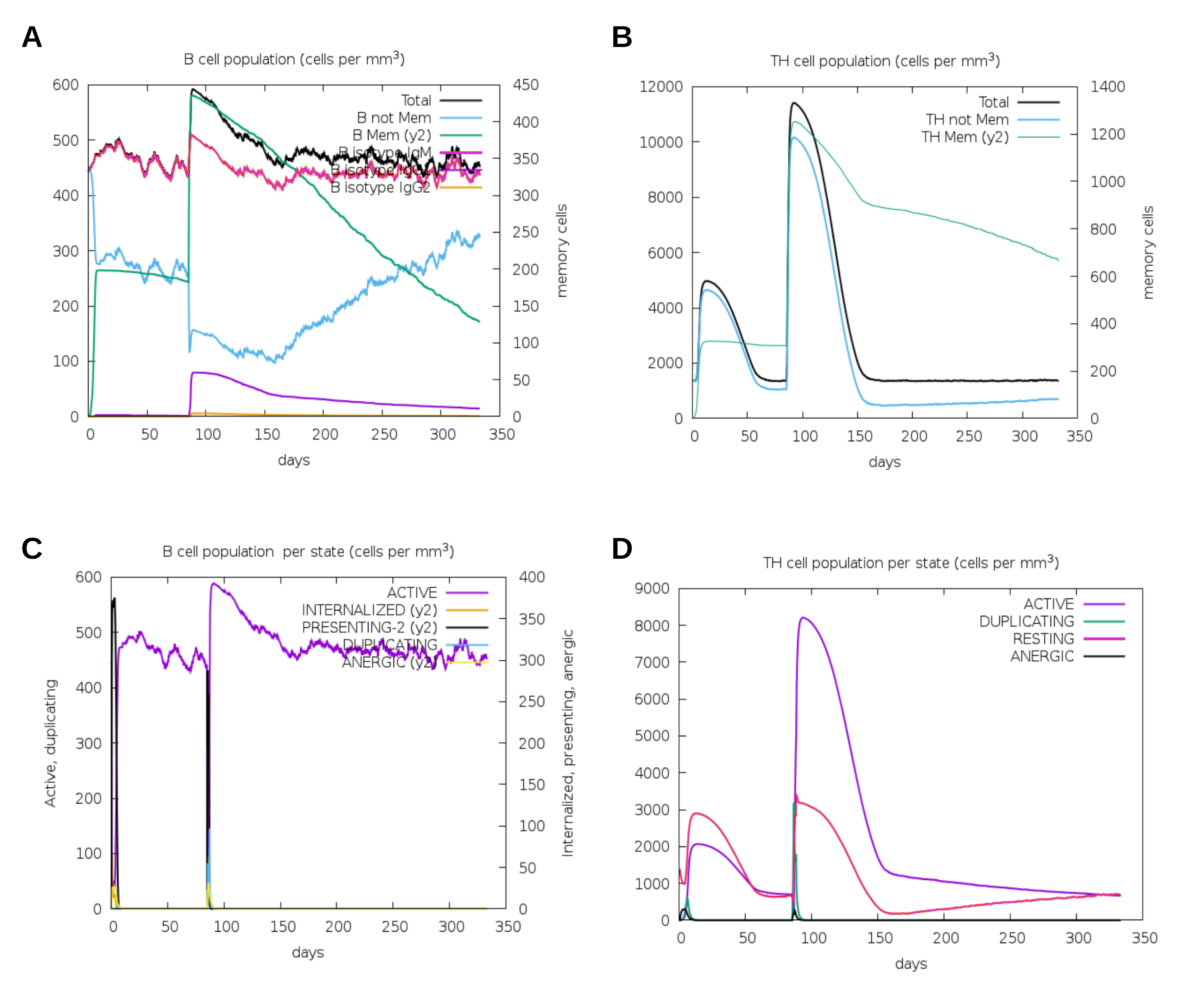

Supplement: Supplemental Information 5 — (A) Concentration of B cell population, including memory B cell after multi-epitope exposures. (B) Concentration of T-cell population, including memory cell after multi-epitope exposures. (C) Concentration of B cell population per state - Active, Internalized the Ag, Presentation on MHC II, Duplicating in the mitotic cycle and Anergic (D) Concentration of T cell population per state - Active, Duplicating in the mitotic cycle, Anergic and Resting - not active. For all panels the specific subclasses are indicated as colored peaks. [file peerj-09-12548-s005.png]

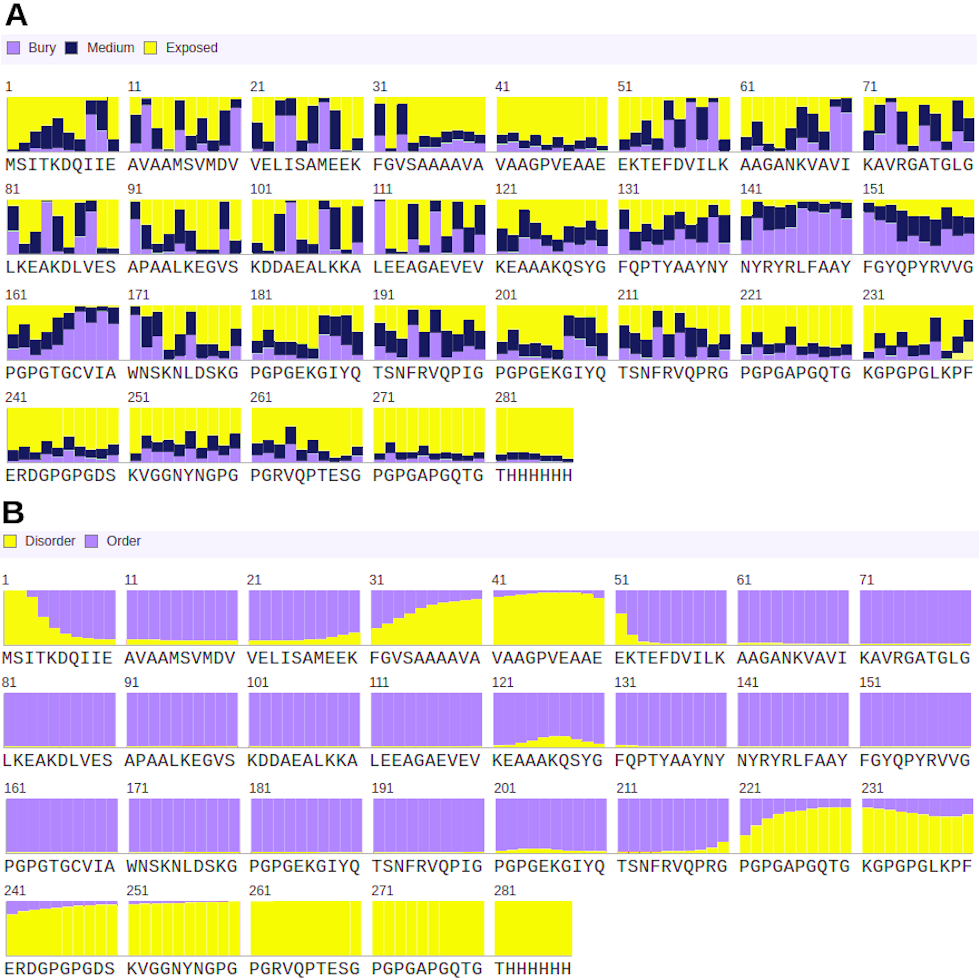

Supplement: Supplemental Information 6 — (A) shows the solvent accessibility of each residue along the structure, and the proportion of buried, medium exposed and high exposed residues. (B) represents the disorder degree of each residue. [file peerj-09-12548-s006.png]

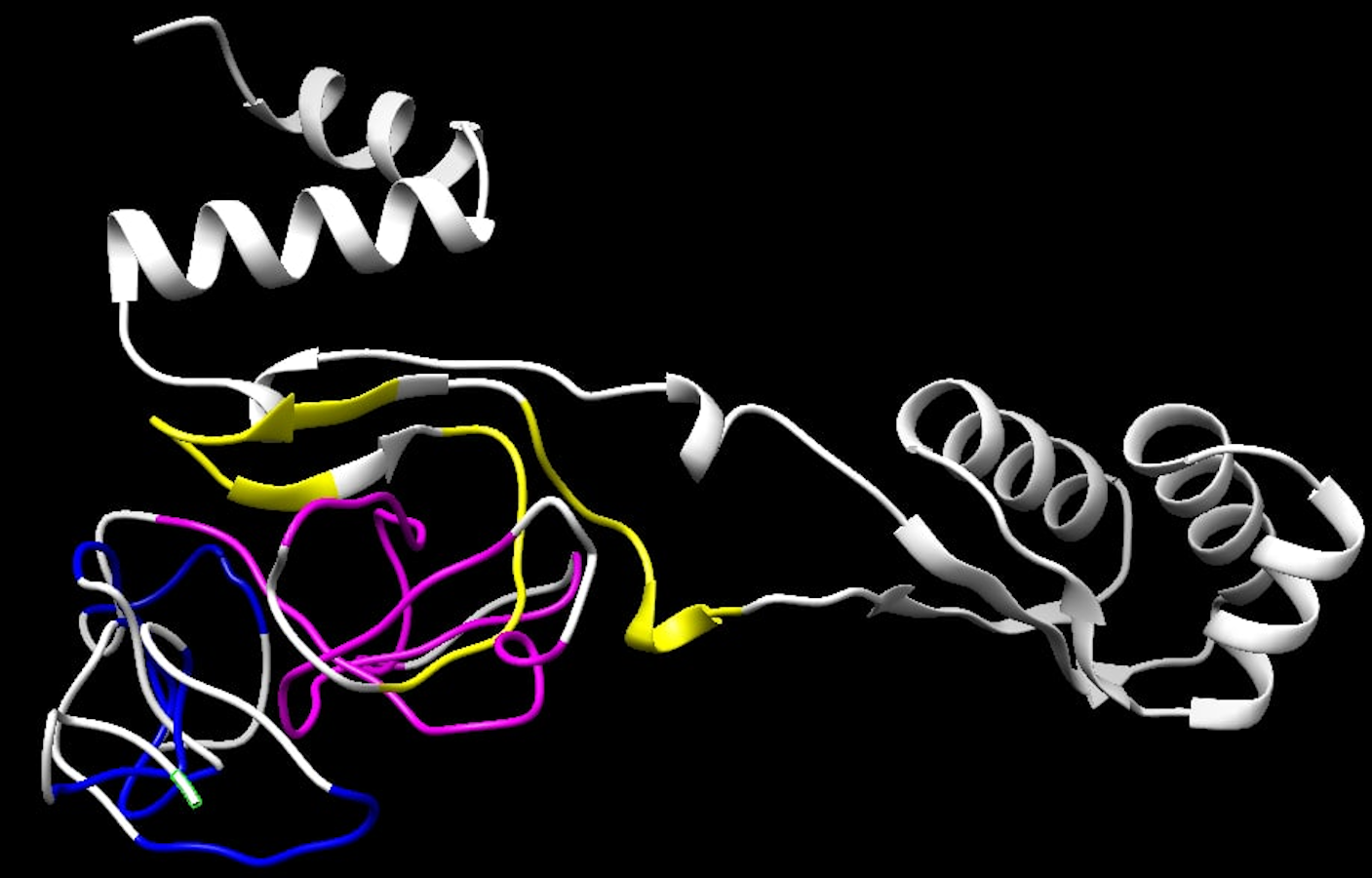

Supplement: Supplemental Information 7 — The structure refined using the GalaxyRefine server had all the RBD epitopes highlighted following their type: yellow color refers to HLA class I T-cell epitopes, pink color corresponds to the HLA class II T-cell epitopes, and the B-cell epitopes are represented by the blue color. [file peerj-09-12548-s007.png]

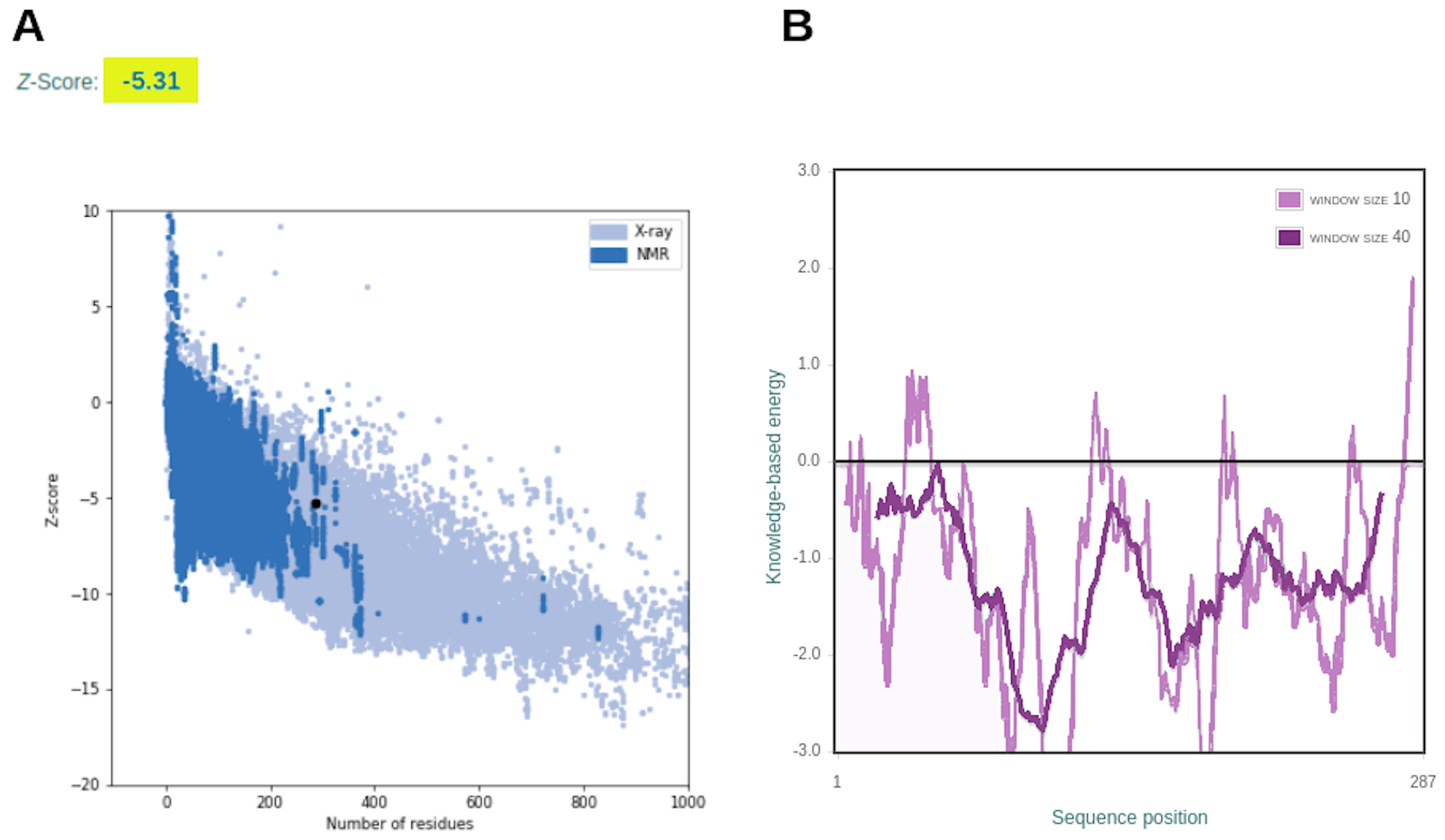

Supplement: Supplemental Information 8 — (A) shows the z-score comparing our multi-epitope structure to determined structures from PDB of the same size. (B) corresponds to the level of energy of each residue. [file peerj-09-12548-s008.png]

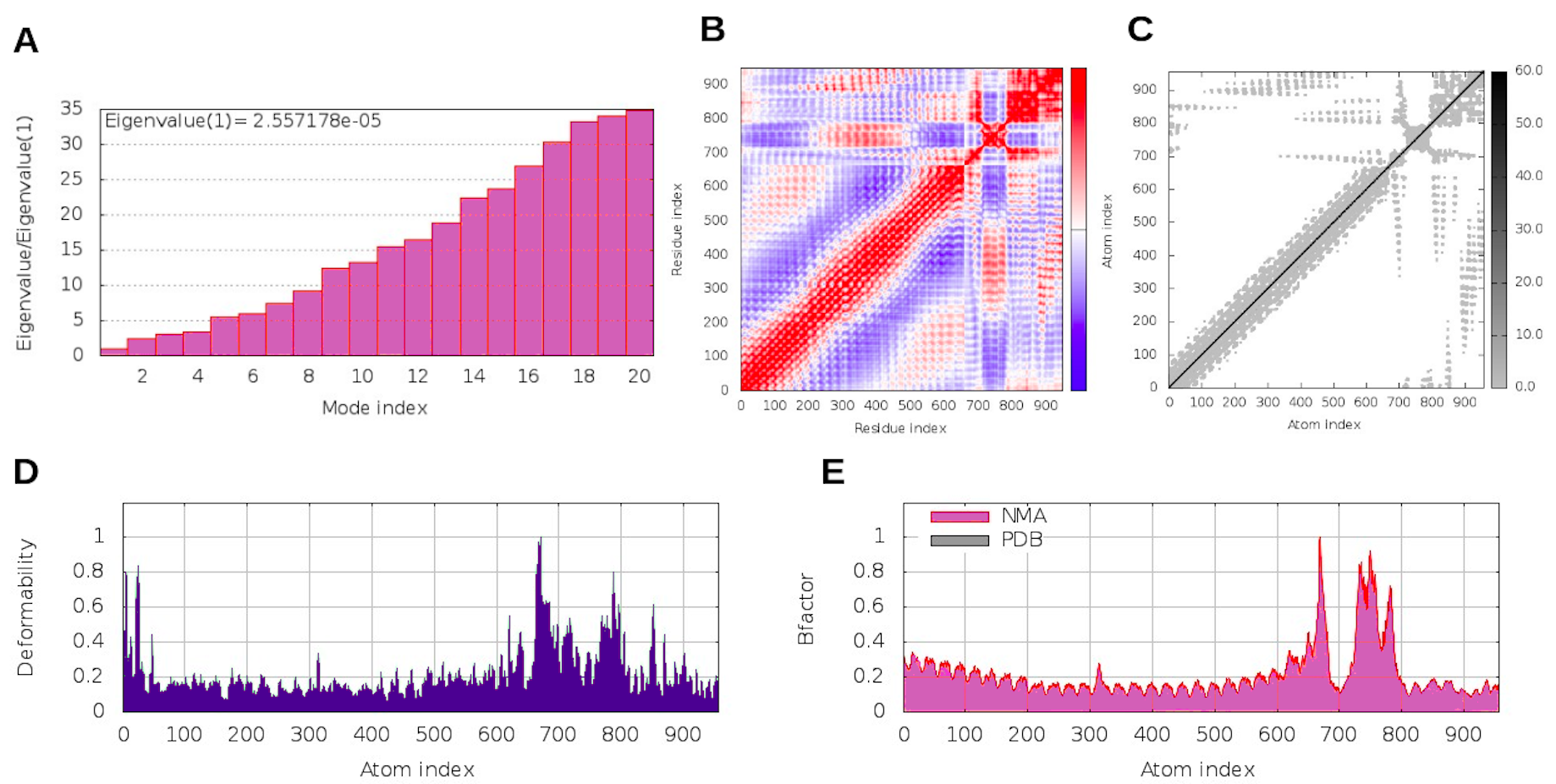

Supplement: Supplemental Information 9 — (A) refers to the eigenvalue and the needed energy for structure deformation; (B) shows the covariance matrix representing the unrelated (white), correlated (red) and anti-correlated (blue) residues; (C) demonstrates the results of the elastic network model and the darker gray regions point to more rigid springs; plot (D) shows the main-chain deformability simulation; and (E) represents the uncertainty quantification for each residue through the b-factor values. [file peerj-09-12548-s009.png]

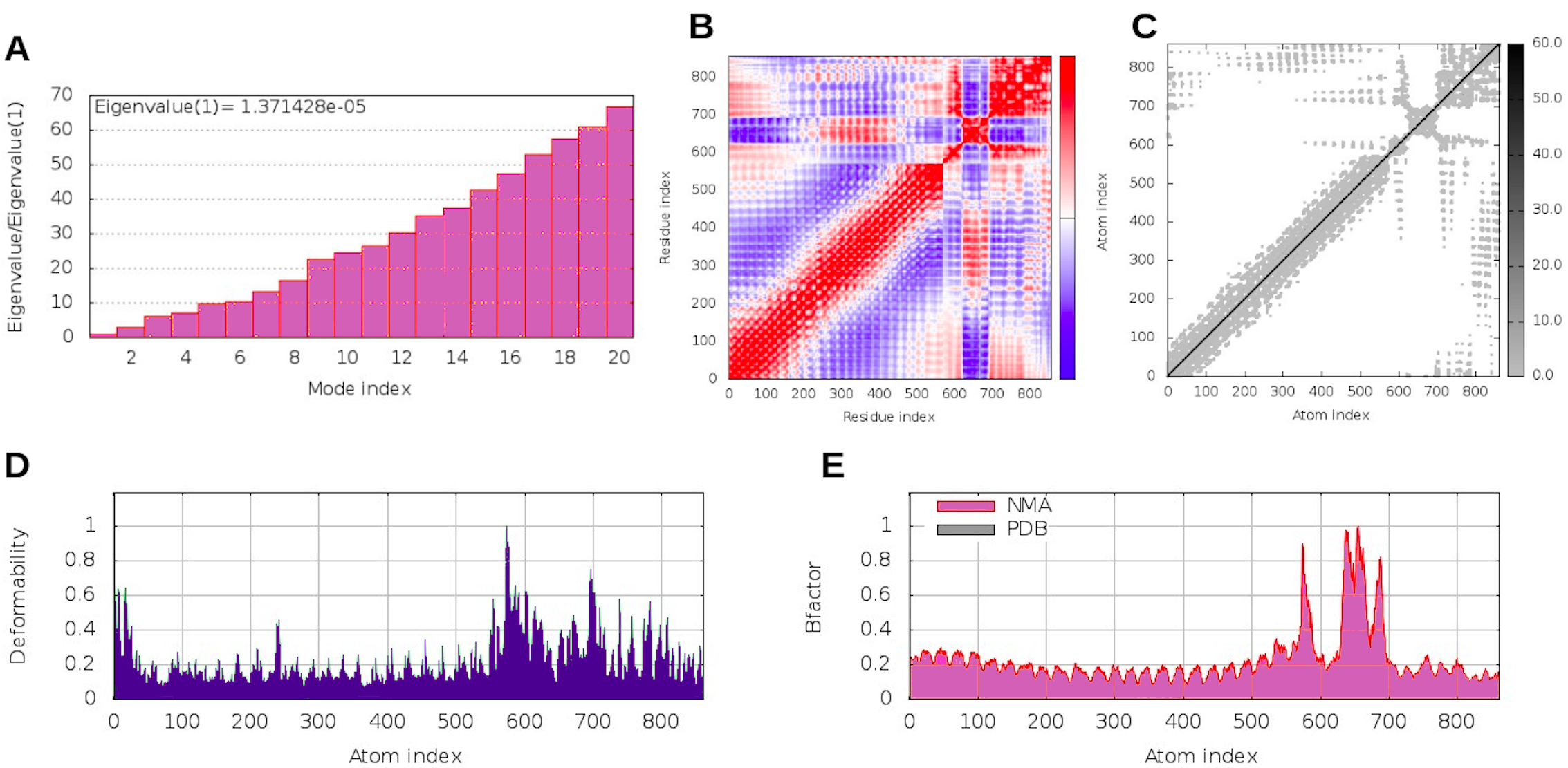

Supplement: Supplemental Information 10 — (A) refers to the eigenvalue and the needed energy for structure deformation; (B) shows the covariance matrix representing the unrelated (white), correlated (red) and anti-correlated (blue) residues; (C) demonstrates the results of the elastic network model and the darker gray regions point to more rigid springs; plot (D) shows the main-chain deformability simulation; and (E) represents the uncertainty quantification for each residue through the b-factor values. [file peerj-09-12548-s010.png]
